# The association of social networks with the job performance of primary health care professionals: the mediating effect of knowledge sharing

**DOI:** 10.3389/fmed.2024.1324939

**Published:** 2024-10-03

**Authors:** Xiubo Wang, Shengchao Hou, Qiongxin Lv, Yuxin Liu, Huan Wu, Zhiyong Liu

**Affiliations:** ^1^School of Medicine and Health Management, Tongji Medical College, Huazhong University of Science and Technology, Wuhan, China; ^2^Tongji Hospital, Tongji Medical College, Huazhong University of Science and Technology, Wuhan, China; ^3^Shenzhen Children’s Hospital, Shenzhen, China; ^4^School of Management, Xinxiang Medical University, Xinxiang, China

**Keywords:** social networks, knowledge sharing, job performance, social media, primary health care professional

## Abstract

**Background and aims:**

Social networks formed through social media platforms have facilitated knowledge sharing among primary health care professionals (PHCPs). However, the impact of these networks on PHCPs’ job performance and the mediating role of knowledge sharing remain underexplored. This study aimed to investigate the association between social networks formed via social media and the job performance of PHCPs, and to explore the mediating role of knowledge sharing in this association.

**Methods:**

A cross-sectional survey was carried out among PHCPs in Henan Province, China, involving 655 valid responses. Validated scales measured the key variables, and structural equation modeling (SEM) tested the proposed hypotheses, including the mediating effect of knowledge sharing through bootstrap method. Statistical analysis was performed using SPSS 24.0 and AMOS 24.0.

**Results:**

The degree centrality (*β* = 0.225; *p* = 0.001) and network heterogeneity (*β* = 0.093; *p* = 0.043) of the social network had a significant direct association with job performance, whereas the direct associations of betweenness centrality and network tie strength with job performance were not significant. Knowledge sharing mediated the relationship between degree centrality (*β* = 0.147; *p* = 0.001), network heterogeneity (*β* = 0.251; *p* = 0.043), and job performance.

**Conclusion:**

The study revealed the internal mechanisms by which social network characteristics influence PHCPs’ job performance, highlighting the mediating role of knowledge sharing. Social networks formed within social media contexts have multifaceted effects on job performance, with knowledge sharing as a critical mediating variable. These findings underscore the importance of leveraging social media for professional networking and knowledge exchange to enhance PHCPs’ job performance.

## 1 Introduction

Primary health care (PHC) entails the provision of integrated and accessible essential healthcare services by healthcare professionals, with a primary focus on addressing the basic health needs of individuals and communities. PHC stands as a key model for promoting public health globally. Notably, in China, the government has accorded high priority to the development of PHC. Since the initiation of comprehensive healthcare reforms in 2009, China has implemented a series of pivotal policies, including the establishment of the national hierarchical medical system, the introduction of medical consortia, and the reinforcement of PHC institutions. These measures were designed to enhance residents’ accessibility to PHC and optimize resource allocation within PHC institutions (1, 2). Despite these comprehensive reforms leading to remarkable improvements in PHC service capacity (3), a persistent gap persists between the intended policy objectives and the actual state of development. This gap primarily arises from the incapacity of primary healthcare professionals (PHCPs) to meet the escalating demand for PHC services among residents (4).

PHCPs occupy a pivotal role as frontline professionals actively engaged in the delivery of PHC services, effectively functioning as the “gatekeepers” of residents’ health. Their performance exerts a decisive influence on the substantial enhancement of PHC service capacity and the successful execution of healthcare reforms. The concept of PHCPs’ job performance encompasses a composite of behaviors, skills, and abilities demonstrated by these professionals in the execution of their duties and responsibilities to achieve organizational objectives within the healthcare sector (5). This construct is influenced by several factors, such as financial incentives, workload, social support, leadership, job satisfaction, and knowledge sharing (6–11). Knowledge sharing, in this context, is defined as the process through which healthcare professionals exchange, disseminate, and co-create knowledge and information within their networks. This includes transferring skills, experiences, and insights through interactions facilitated by social media platforms, which operate on both explicit and tacit levels.

Despite these insights, the impact of organizational attributes on PHCPs’ job performance has often overlooked the critical role played by social networks, which are formed through interactions among employees within organizations. These social networks, integral components of the organizational fabric, have substantial influence over both individual and collective performance (12).

The exploration of social networks and job performance has become inextricably intertwined with the realm of social media in recent times. Social media, defined as a collection of online applications and platforms built upon the ideological and technological foundations of Web 2.0, is intrinsically linked to specific networks of user social connections, enabling users to create, share, and seek content (13–15). In China, widely utilized social media platforms, such as WeChat, Tencent QQ, and DingTalk, play a pivotal role in fostering interpersonal networks (16, 17). A ‘social network’ in this context refers to the structure of relationships and interactions among individuals, specifically health care professionals, which are facilitated by digital platforms. These networks are characterized by nodes (individual actors, people, or things within the network) and ties (the relationships or interactions between these actors). Functionally, social networks facilitate the sharing of information, collaboration, and peer support among professionals, thereby enhancing communication and coordination within healthcare settings.

Before COVID-19, social media adoption in healthcare was steadily growing due to its potential for enhancing communication and collaboration. The pandemic has accelerated this trend, making social media vital for maintaining professional networks and exchanging critical medical information. With face-to-face interactions limited, social media has become essential for sustaining social networks among PHCPs, facilitating efficient knowledge exchange. This increased reliance highlights the need to explore how these networks impact PHCPs’ job performance, especially in a rapidly evolving digital healthcare environment (18, 19). However, research addressing the impact of social networks on PHCPs’ job performance within the context of social media environments remains limited, with the underlying mechanisms of this relationship still unclear.

In consideration of these factors, this study endeavors to investigate how the structural and relational characteristics of PHCPs’ social networks via social media influence their job performance. Furthermore, the study will delve into the role of knowledge sharing as an intermediary variable within this relationship, illuminating the intricate mechanisms that underscore the effect of social network characteristics on job performance within the context of social media. The results of this study can provide a reference for PHC institutions to create an organizational environment conducive to better interpersonal synergies and high-quality healthcare service.

## 2 Research model and hypothesis development

### 2.1 Social networks and job performance

The social network is a collection of relationships including kinship, friendship, and superior-subordinate relationships in an organization (20, 21). Social media is a popular tool utilized by health care professionals to meet their professional needs, creating a social network that is more accessible than engaging in face-to-face interactions (22). As indicated by Burt and Granovetter’s study, social networks can be conceptualized in terms of two dimensions: structure and relationships (23, 24). In this study, the structural dimension considers network centrality and heterogeneity, while the relational dimension is assessed through network tie strength.

Network centrality is used to represent the overall structure of social networks and can be measured by degree centrality and betweenness centrality (25–27). In the context of healthcare, degree centrality refers to the number of direct connections that a healthcare worker has with other workers within the healthcare system. Betweenness centrality measures the extent to which a healthcare worker lies on the shortest path between other pairs of workers within the healthcare system. According to social capital theory, a higher degree centrality and betweenness centrality leads to greater access to social benefits in the network, facilitating problem solving and relationship building at work and thereby improving individual performance (28). Previous studies have demonstrated positive relationships between the network centrality of personal networks and individual performance (29–32). PHCPs with high degree centrality usually have enhanced connectivity and access to valuable resources and information. PHCPs with high betweenness centrality act as intermediaries and can facilitate communication and collaboration among different parts of the healthcare system, which can lead to more effective teamwork and better patient outcomes.

Network heterogeneity refers to the number and types of differences that exist between PHCPs in a given social network (33, 34). The use of social media tools enables healthcare professionals to more easily establish connections and expand their network heterogeneity. This can positively influence their job performance, as they are more likely to receive support from individuals with diverse perspectives, experiences, and knowledge backgrounds, thus fostering their confidence in delivering high-quality patient care.

Network tie strength refers to the frequency of contact and emotional closeness between PHCPs in a social network (24, 35, 36). It is noteworthy that in Chinese guanxi society, strong ties play a more important role than weak ties in facilitating collaboration and information exchange among individuals (37). Connections via social media between PHCPs and their colleagues can make the transmission of information faster and more extensive, leading to enhanced job satisfaction and increased work efficiency and ultimately resulting in better performance.

Based on the above analysis, we hypothesize as follows:

*H1:* Social networks of PHCPs have a significant positive effect on job performance.

*H1a:* The degree centrality of PHCPs has a significant positive effect on job performance.

*H1b:* The betweenness centrality of PHCPs has a significant positive effect on job performance.

*H1c:* The network heterogeneity of PHCPs has a significant positive effect on job performance.

*H1d:* The network tie strength of PHCPs has a significant positive effect on job performance.

### 2.2 Social networks and knowledge sharing

Knowledge sharing represents a dynamic process through which knowledge is transmitted from the knowledge owner to the recipient, subsequently assimilated, and internalized by the latter (38, 39). It is noteworthy that social networks can serve as potent motivators for knowledge sharing endeavors (40). The advent of social media platforms has greatly facilitated the practice of knowledge sharing (41). Individuals’ proclivity for knowledge sharing is significantly influenced by their roles and positions within their respective social networks. Specifically, PHCPs occupying pivotal positions characterized by high degree and betweenness centrality are more inclined to partake in knowledge sharing activities within the confines of their social networks. PHCPs with a high degree centrality, owing to their extensive network connections, play a particularly advantageous role in sharing medical knowledge and responding to requests for assistance from colleagues. On the other hand, PHCPs with high betweenness centrality, a key attribute for efficiently diffusing and regulating information across diverse teams, are motivated by the desire to maintain their network leadership status, leading them to actively participate in the dissemination of knowledge (42).

Network heterogeneity serves as a catalyst for promoting knowledge sharing among PHCPs by facilitating diverse knowledge exploration (43). Social media platforms act as converging arenas for healthcare practitioners, encompassing a diverse array of backgrounds and geographical origins, each contributing unique expertise and experiential insights (14, 44). This convergence offers PHCPs convenient access to a multitude of novel ideas and information from disparate sources, thereby stimulating their proclivity for knowledge sharing. The intrinsic diversity within these networks possesses the inherent capability (45) to augment individual cognitive resources. In contrast to interactions with peers possessing similar knowledge and experiences, engagement with individuals characterized by contrasting knowledge and diverse experiences introduces PHCPs to a rich tapestry of innovative ideas and varying viewpoints. This, in turn, fosters a dynamic exchange of knowledge.

Network tie strength may have a positive impact on knowledge sharing. This phenomenon is particularly evident in the context of tacit knowledge exchange (46). Frequent social media interactions between PHCPs have been found to enhance the maintenance of close relationships while simultaneously promoting trust and collaboration (47). This, in turn, facilitates the dissemination of valuable information and enhances it, thereby augmenting the overall knowledge sharing process.

Thus, we hypothesize the following:

*H2:* Social networks of PHCPs have a significant positive effect on knowledge sharing.

*H2a:* The degree centrality of PHCPs has a significant positive effect on knowledge sharing.

*H2b:* The betweenness centrality of PHCPs has a significant positive effect on knowledge sharing.

*H2c:* The network heterogeneity of PHCPs has a significant positive effect on knowledge sharing.

*H2d:* The network tie strength of PHCPs has a significant positive effect on knowledge sharing.

### 2.3 Social networks and job performance

Knowledge sharing is a crucial and valued social asset for organizations since it leads to improved job performance and enhances organizational success, particularly in social media environments (48, 49). Both explicit and tacit knowledge are shared to enhance individual and organizational performance by interrelating learning, practice, and peer input (50). Previous research has indicated that knowledge sharing promotes better job skills, greater job knowledge, and increased work efficiency, consequently leading to enhanced job performance (51–53). As a typical knowledge-intensive organization, PHCIs depend on knowledge sharing and learning innovation among PHCPs to enhance their core competitiveness and improve the quality of healthcare services. Several studies have shown that sharing knowledge, including clinical knowledge, work skills, and experience, is conducive to the enhancement of clinical diagnosis and decision-making, as well as the optimization of healthcare services (54). Therefore, we hypothesize the following:

*H3:* Knowledge sharing among PHCPs has a significant positive effect on job performance.

By synthesizing the analysis of both hypotheses 2 and 3, it is reasonable to further propose that knowledge sharing acts as a mediator between the social networks of PHCPs via social media and their job performance.

*H4:* Knowledge sharing has a significant mediating effect between PHCPs’ social networks and job performance.

*H4a:* Knowledge sharing has a significant mediating effect between degree centrality and job performance.

*H4b:* Knowledge sharing has a significant mediating effect between betweenness centrality and job performance.

*H4c:* Knowledge sharing has a significant mediating effect between network heterogeneity and job performance.

*H4d:* Knowledge sharing has a significant mediating effect between network tie strength and job performance.

**Figure 1:**
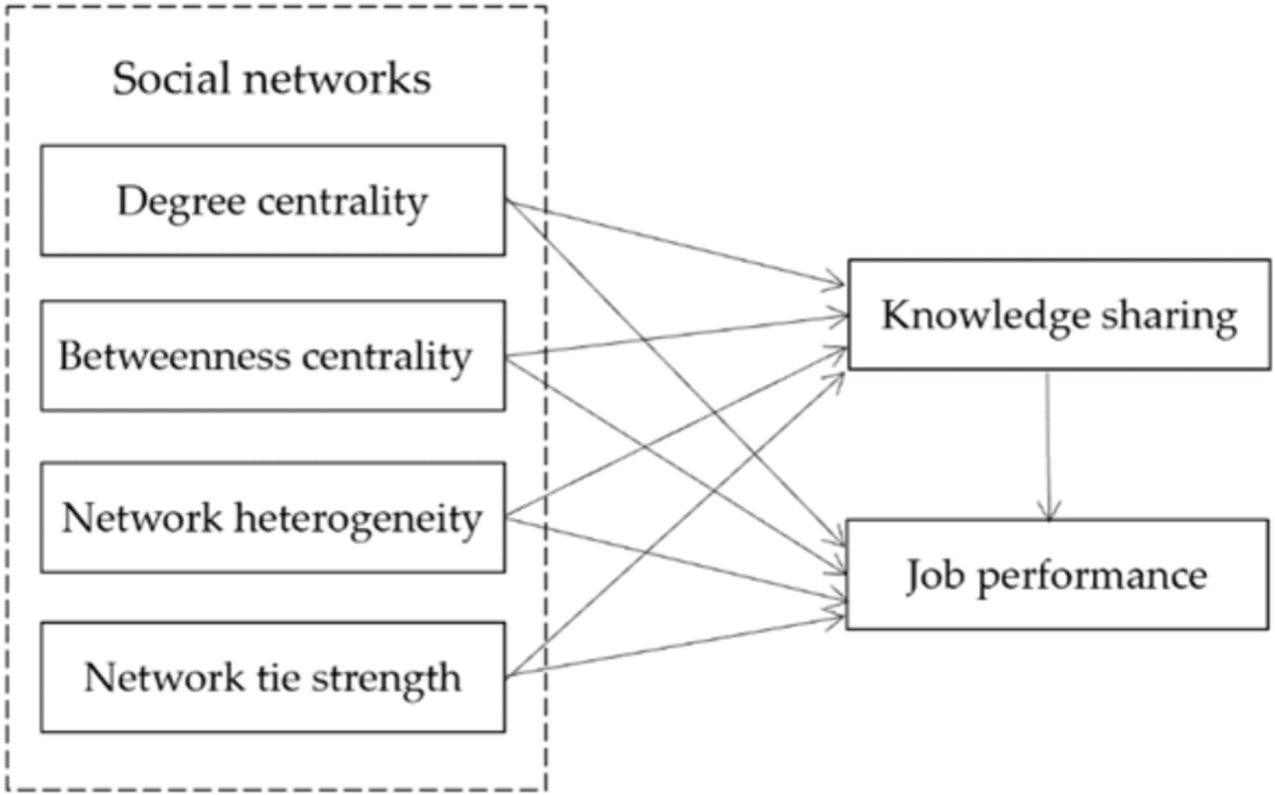
Research model.

Based on the analysis above, the hypothesized research model was developed and is presented in Figure 1. This research model investigates the relationships between social networks, knowledge sharing, and job performance among PHCPs. Specifically, it posits that degree centrality, betweenness centrality, network heterogeneity, and network tie strength directly influence job performance, with these effects being further mediated by knowledge sharing. By integrating social network theory and knowledge-sharing theory, this study aims to explore how the structural and relational characteristics of PHCPs’ social networks within a social media environment impact their job performance. The model underscores the essential role of knowledge exchange as a mechanism for enhancing professional performance, thereby providing insights into the ways that effective collaboration and knowledge sharing can lead to improved outcomes in primary health care settings.

## 3 Methodology

### 3.1 Measurement instrument

The questionnaire used in this study was obtained from a reliable source with proven validity and reliability. In this study, social networks are divided into four latent variables: degree centrality, betweenness centrality, network tie strength, and network heterogeneity. According to Freeman’s (55) research, four measurement items for degree centrality and three for betweenness centrality were developed. Network heterogeneity is assessed using the five measurement items originally proposed by Marsden (56) and Harrison (57). Moreover, network tie strength is measured using three items adapted from the studies of Burt (23) and Granovetter (24). To measure knowledge sharing, this study references the scale developed by Bock (39, 58), comprising six items, specifically three for explicit knowledge sharing and three for tacit knowledge sharing. Additionally, the job performance scale was developed based on the previous research of Borman and Motowidlo (59, 60) and Van Scotter and Motowidlo (61), covering dimensions of task performance, interpersonal facilitation, and job dedication. Each dimension is assessed using three items. A pretest of the questionnaire is conducted with three experts to assess logical consistency, ease of comprehension, adequacy of question item sequence, and contextual appropriateness. For specific item details, please see Supplementary Appendix 1. All the latent variables are measured using a five-point Likert scale, ranging from “strongly disagree” to “strongly agree.”

### 3.2 Data collection

To enhance the validity of the research instrument, a presurvey was conducted. Based on the results of reliability and validity tests, the scale items were refined, leading to the development of the final questionnaire. The survey was administered to PHCPs in a pilot city of a medical consortium in Henan Province, China, from October 7, 2021, to October 30, 2021. The convenience sampling was chosen due to its practicality and feasibility within the context of the study. We selected participants from a diverse range of community health centers, township health centers, and village clinics to maximize the representativeness of the sample. The survey was distributed through the WenJuanXing platform using an online questionnaire. This platform not only made it convenient for participants to respond to the questionnaire but also ensured the integrity of the data by requiring all questions to be answered before submission. This feature prevented incomplete submissions and eliminated the possibility of resubmission, thereby maintaining the quality and integrity of the responses. Out of the 726 questionnaires distributed to participants, 655 were identified for inclusion in the study and used for analysis (participation rate 90.2%). The use of convenience sampling, although not probabilistic, aimed to capture a broad spectrum of PHCPs’ experiences and perspectives, which we believe contributes to the study’s generalizability within the specific context of the medical consortium in Henan Province. This survey was approved by the Ethics Committee of the Xinxiang Medical University (reference number XYLL-2,017,032).

### 3.3 Data statistical analysis

The collected data were analyzed using statistical techniques, including descriptive statistics, confirmatory factor analysis (CFA), and structural equation modeling (SEM). The initial step involved exploring participant characteristics such as gender, age, educational background, and years of experience using the descriptive statistics tools provided by the SPSS software.

A CFA was conducted in the second step to determine the optimal measurement model. The model’s reliability was assessed through calculations of Cronbach’s alpha coefficient and the composite reliability (CR) values. The factor loadings and average variance extracted (AVE) were used to evaluate the convergent validity of the measurement model.

Subsequently, SEM was conducted using IBM SPSS Amos 22 software to examine the underlying assumptions in a structural model. SEM considers both observed and latent variables, providing a more comprehensive understanding of the interconnections between them. The fit of the SEM was evaluated through the use of various fit indices, including the χ^2^/df-ratio, root mean square error of approximation (RMSEA), goodness-of-fit index (GFI), adjusted goodness-of-fit (AGFI), comparative fit index (CFI), incremental fit index (IFI), and Tucker–Lewis index (TLI). These indices are chosen for their ability to provide a nuanced assessment of the accuracy and meaningfulness of the SEM results, ensuring that the model’s fit is rigorously evaluated from multiple perspectives. Furthermore, Within the framework of SEM, path coefficients were estimated, a process essential for delineating the relationships between variables within the research model. The estimation of these coefficients is a fundamental feature of SEM, justifying its application for inferring the strength and direction of relationships as per the theoretical framework. In aggregate, the utilization of SEM, complemented by descriptive statistics and confirmatory factor analysis (CFA), was selected to enable an exhaustive exploration of the relationships among variables. This methodological approach ensures robust and reliable findings, fitting the intricate nature of the research questions and providing a solid justification for the analytical techniques employed.

## 4 Results

### 4.1 Data collection

Demographic information for the sample of 655 participants is presented in Table 1. The sample consisted of 471 females (71.9%) and 184 males (28.1%). Within this group, 30.7% were aged 31 to 40, while 32.8% fell into the age range of 41 to 50. In terms of education, 64.3% had attained junior college level or below, while 33.4% held a bachelor’s degree. Additionally, 40.8% of respondents had worked for 20 years or more. Based on the assessments from the health department, three levels of professional titles (i.e., junior, intermediate, and senior) are used to denote PHCPs’ proficiency and seniority. Junior-level professional titles were held by 36.6% of PHCPs, with intermediate-level titles held by 29.8% and only 6.3% holding senior or above professional titles. In regard to social media usage, WeChat, a mainstream platform, was the most commonly used at 98%. The intra-organiz,ational social media platform was used at a rate of 25.5%, while the usage rate of other media was relatively low. It is worth noting that DingTalk is gradually attracting the attention of PHCIs.

**Table 1:** Demographic information.

Characteristics	Items	Statistics, *n* (%)
Gender	Male	184 (28.1)
	Female	471 (71.9)
Age (years)	<25	32 (4.9)
	26–30	100 (15.3)
	31–40	201 (30.7)
	41–50	215 (32.8)
	>50	107 (16.3)
Education	Junior college or below	421 (64.3)
	Bachelor’s degree	219 (33.4)
	Master’s degree and above	15 (2.3)
Years of experience	<1	12 (1.8)
	1–3	51 (7.8)
	4–10	139 (21.2)
	11–20	186 (28.4)
	>20	267 (40.8)
Professional title	No title	179 (27.3)
	Junior	240 (36.6)
	Intermediate	195 (29.8)
	Senior	41 (6.3)
Frequently used social media at work (Multiple choice)	WeChat	642 (98)
	QQ	131 (20)
	Weibo	19 (2.9)
	Intra-organizational social media platform (e.g., DingTalk)	167 (25.5)
	Others	13 (2)

### 4.2 Reliability and validity

The Cronbach’s alpha coefficients for the scales of the social network, knowledge sharing, and job performance are all above 0.8. Additionally, Table 2 presents Cronbach’s alpha coefficients and CR values for each intrinsic dimension of the three scales, which are also above 0.8, indicating that the scales exhibit high levels of internal consistency and reliability. The items’ factor loadings were all above 0.6, and AVE for each construct was also above 0.6, demonstrating good convergent validity in the scales. Table 3 shows that the square root of each variable’s AVE value is greater than its correlation coefficient with other dimensions, indicating satisfactory discriminant validity. The correlations between the variables were analyzed, and it was found that there was no high correlation between the variables, as shown in Table 3. Hence, the survey instrument has good reliability and validity.

**Table 2:** Construct reliability and convergent validity.

Variable	Construct	Items	Factor loading	Average variance extracted	Composite reliability	Cronbach’s alpha
Social networks	Degree centrality	DC1	0.885	0.698	0.902	0.900
		DC2	0.907			
		DC3	0.813			
		DC4	0.726			
	Betweenness centrality	BC1	0.796	0.699	0.874	0.871
		BC2	0.904			
		BC3	0.803			
	Network heterogeneity	NH1	0.811	0.737	0.933	0.933
		NH2	0.862			
		NH3	0.875			
		NH4	0.887			
		NH5	0.854			
	Network tie strength	NTS1	0.859	0.718	0.884	0.883
		NTS2	0.892			
		NTS3	0.788			
Knowledge sharing	Explicit knowledge sharing	EKS1	0.875	0.790	0.918	0.919
		EKS2	0.893			
		EKS3	0.898			
	Tacit knowledge sharing	TKS1	0.944	0.888	0.960	0.966
		TKS2	0.947			
		TKS3	0.935			
Job performance	Task performance	TP1	0.867	0.792	0.919	0.918
		TP2	0.925			
		TP3	0.876			
	Interpersonal facilitation	IF1	0.901	0.847	0.943	0.942
		IF2	0.904			
		IF3	0.955			
	Job dedication	JD1	0.883	0.844	0.942	0.940
		JD2	0.963			
		JD3	0.909			

DC, degree centrality; BC, betweenness centrality; NH, network heterogeneity; NTS, network tie strength; EKS, explicit knowledge sharing; TKS, tacit knowledge sharing; TP, task performance; IF, interpersonal facilitation; JD, Job dedication.

**Table 3:** Discriminant validity.

	DC	BC	NH	NTS	EKS	TKS	TP	JD	IF
DC	0.836								
BC	0.380	0.836							
NH	0.278	0.387	0.858						
NTS	0.483	0.485	0.439	0.847					
EKS	0.360	0.460	0.356	0.462	0.889				
TKS	0.367	0.396	0.343	0.484	0.750	0.942			
TP	0.317	0.213	0.277	0.263	0.299	0.333	0.89		
JD	0.302	0.154	0.178	0.220	0.250	0.326	0.682	0.919	
IF	0.341	0.241	0.247	0.302	0.317	0.367	0.774	0.751	0.92

*p < 0.05; **p < 0.01; ***p < 0.001. KS, knowledge sharing; JP, job performance.

This study utilized Harman’s single-factor test (62) to perform an exploratory factor analysis on the survey data. The first factor explaining 36.218% of the variance, less than 40%. Thus, it can be inferred that common method bias did not significantly affect the sample data collected in this study.

Table 4 shows that the model fits the data well, with a χ^2^/df-ratio of less than 3 and an RMSEA of under 0.05, indicating reliability and robustness. In addition, the GFI, AGFI, CFI, IFI, and TLI all exceeded 0.9, suggesting a good fit between the model and the data. These high initial fit indices further support the model’s suitability for explaining the observed relationships among the variables.

**Table 4:** Goodness-of-fit indices of the structural equation model.

Indices	*χ*^2^/df	RMSEA	GFI	AGFI	CFI	IFI	TLI
Criteria	<3	<0.05	>0.9	>0.9	>0.9	>0.9	>0.9
Value	2.232	0.043	0.917	0.900	0.974	0.974	0.970

### 4.3 Path analysis

**Figure 2:**
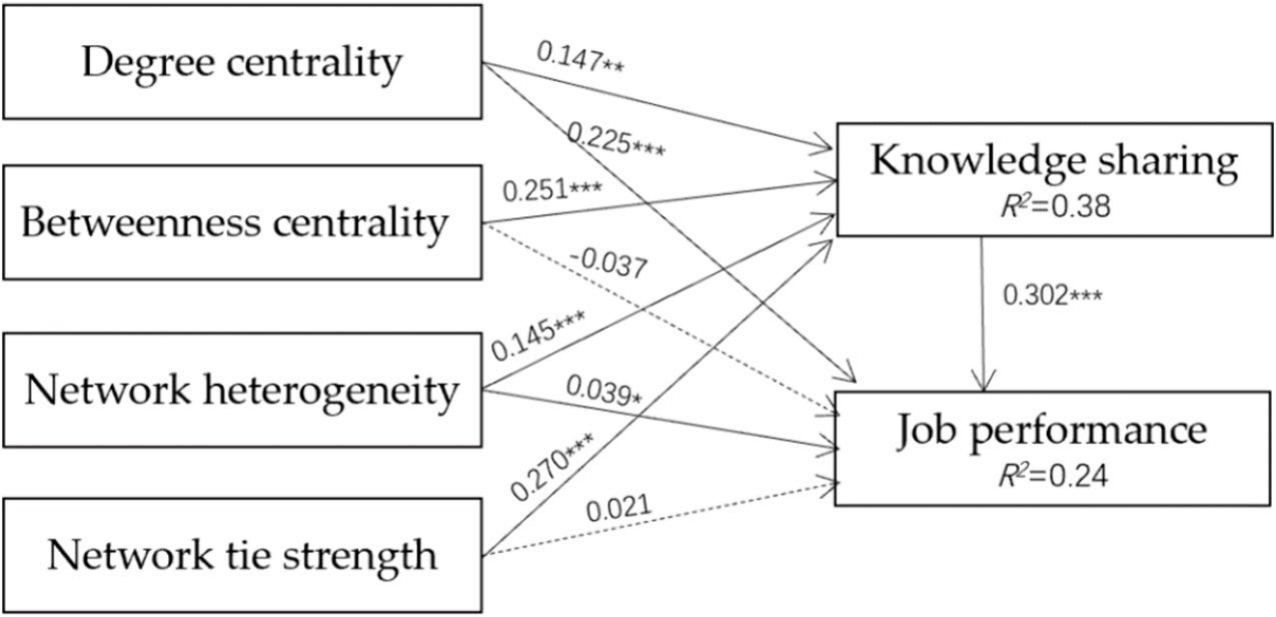
SEM statistical model. *p < 0.05; **p < 0.01; ***p < 0.001.

Figure 2 shows that social networks impacted knowledge sharing significantly. The unstandardized regression coefficients from degree centrality, betweenness centrality, network heterogeneity, and network tie strength to knowledge sharing were 0.147, 0.251, 0.145, and 0.27, respectively. All the *p* values were less than 0.001, which meant that social networks impacted knowledge sharing significantly. Degree centrality, network heterogeneity, and knowledge sharing significantly influenced job performance, with coefficients of 0.225, 0.039, and 0.032, respectively. However, betweenness centrality and network tie strength had no significant influence on job performance.

This study used bootstrapping as the repeated sampling method to produce statistical confidence intervals for indirect effects. Table 5 displays the statistically significant indirect effects of knowledge sharing in the paths from degree centrality, betweenness centrality, network heterogeneity, and network tie strength to job performance. For all four paths, the values of the lower bound and upper bound of the confidence intervals were not across 0, which meant that an indirect effect existed.

**Table 5:** Indirect effects of the estimated SEM using bootstrapping (2000 replications).

Indirect effects	Product of coefficients			Bootstrapping procedure
	Est.	S.E.	*p* Value	CI 95%
DC→KS→JP	0.031	0.015	0.006	(0.009, 0.069)
BC→KS→JP	0.041	0.013	0.000	(0.021, 0.076)
NH→KS→JP	0.032	0.016	0.010	(0.008, 0.071)
NTS→KS→JP	0.064	0.026	0.001	(0.026, 0.134)

## 5 Discussion

### 5.1 Principal findings and explanations

Our study reveals the significant influence of social networks on knowledge sharing and job performance among PHCPs in China. Specifically, degree centrality and network heterogeneity were found to have substantial effects on knowledge sharing, which in turn positively impacts job performance. This finding aligns with a recent study on hypertension management teams, which demonstrated that in-closeness centrality of advice networks positively affects task performance (12). Interestingly, while betweenness centrality and network tie strength did not directly impact job performance, they significantly influenced knowledge sharing. This relationship highlights the complex nature of social networks in healthcare settings, where different dimensions of centrality contribute to various aspects of professional interactions. Recent research suggests that betweenness centrality is a key measure of node importance in networks (63), which in our context, corresponds to the crucial role certain PHCPs play in facilitating knowledge flow.

Our study confirms that knowledge sharing mediates the relationship between social networks and job performance. This finding is consistent with research demonstrating that knowledge integration is significantly related to patient-centered teamwork and team performance in healthcare settings (64). The mediating role of knowledge sharing underscores its importance as a linking mechanism between the structural attributes of social networks and job performance within PHC institutions. PHCPs who actively engage in knowledge sharing are more likely to excel in their roles, highlighting the necessity of encouraging and facilitating knowledge exchange within healthcare settings (65). This aligns with previous research emphasizing the importance of fostering a knowledge-sharing culture in healthcare organizations (66).

The cultural context of Chinese society profoundly shapes the dynamics of social networks and knowledge sharing among PHCPs. The principles of collectivism, the importance of relational values (Guanxi), and the concept of face (social reputation and dignity) are deeply embedded in professional and social behaviors (67). In a collectivist culture, the emphasis on group harmony and team cooperation is paramount, which may explain the strong influence of degree centrality on knowledge sharing and job performance (68). Our findings resonate with research that found cultural contingencies significantly influence knowledge seeking and providing behaviors (69). PHCPs with high degree centrality, who maintain closer relationships with a broader array of colleagues, are likely to be more actively involved in various tasks. This increased involvement allows them to access diverse information and resources, optimizing workload distribution and task coordination. Their prominent network position also enhances their social reputation or “face,” fostering a supportive and cooperative environment among colleagues. Consequently, this culturally embedded degree centrality enhances individual efficiency, integrates resources, promotes knowledge sharing, and subsequently improves overall job performance.

The study highlights the predominant use of WeChat and DingTalk among PHCPs for social media communication. WeChat, with a 98% usage rate, is favored for its ubiquity in daily life, facilitating informal communication and tacit knowledge exchange. Its real-time, flexible communication features are crucial for sharing experiences, discussing complex clinical information, and collaborating on patient cases. In contrast, DingTalk, used by 25.5% of PHCPs, is better suited for formal knowledge dissemination. Its enterprise features support structured communication, training, and standardized information sharing, making it valuable for formal documentation and organized knowledge transfer. These platforms foster proactive and spontaneous knowledge sharing behaviors, which in turn contribute positively to patient well-being. This dual platform approach aligns with findings on the adoption of social media by clinicians for professional knowledge sharing and social networking (38). Emerging technologies such as artificial intelligence, augmented reality, and blockchain have the potential to enhance communication, improve data security, and facilitate more dynamic and interactive knowledge sharing among healthcare professionals (70). These advancements could further strengthen social networks by making them more efficient and resilient, ultimately improving the job performance of PHCPs.

### 5.2 Theoretical and practical implications

This study enhances social network theory and knowledge management literature in healthcare by revealing the complex interplay between social network centrality and job performance through knowledge sharing, particularly in the context of Chinese primary healthcare, highlighting the crucial role of knowledge exchange mechanisms in driving professional performance. Thereby bridging theoretical understanding with practical implications. The findings of this study carry practical significance for primary healthcare managers and policymakers. To enhance the job performance of PHCPs, several managerial actions could be considered. First, healthcare organizations should actively promote the development of strong social networks among PHCPs, acknowledging the importance of guanxi (social connections) within the Chinese context. This can be facilitated through team-building activities (71), collaborative projects (72), and the utilization of social media platforms that support professional networking. Second, a culture that encourages and rewards knowledge sharing (73) among PHCPs should be cultivated. Barriers to social media-based knowledge sharing, such as knowledge codification costs, fear of losing intellectual capital, and challenges in building digital trust need to be addressed (41). To ensure sustainability and effectiveness, healthcare organizations can implement incentive mechanisms that emphasize both individual and organizational benefits, provide training on digital literacy for effective knowledge-sharing practices, and invest in user-friendly social media tools to enhance participation in knowledge exchange activities. Third, organizations should balance degree centrality and network heterogeneity, as an overemphasis on centralization may lead to information bottlenecks (74). Managers should aim to create networks that balance centrality and diversity. Given the widespread use of platforms such as WeChat and DingTalk among Chinese PHCPs, integrating these platforms into knowledge-sharing strategies is recommended due to their effectiveness in facilitating communication and collaboration (75). Lastly, continuous investment in training and career development opportunities for PHCPs is crucial (76). An effective approach would be to integrate online learning with social media, thereby enhancing the overall knowledge quality of the PHCP social network.

### 5.3 Limitations and future directions

This study has several limitations. First, it relied on subjective self-assessments, potentially introducing bias. Future research should incorporate objective data and include evaluations from colleagues or superiors to enhance cross-validation. Second, the use of cross-sectional data constrains causal inference. Future studies should employ longitudinal or panel data to elucidate causal mechanisms more effectively. Third, the sample was limited to a single city in Henan Province, affecting generalizability. Future research should adopt multicenter designs and larger, more diverse samples to improve the representativeness and applicability of the findings. Fourth, this study examined social networks’ impact on job performance from a static perspective, disregarding their dynamic nature and sustainability of knowledge-sharing over time. Future research should employ dynamic network analysis to capture the evolution of social network structures and their effects on knowledge sharing and job performance. Lastly, this study did not address factors such as information overload, ethical risks, trust within social networks, and the digital literacy and leadership of primary healthcare professionals due to its specific research focus. However, the rapid advancement of internet technology and social media in healthcare will bring significant changes and challenges. Future research should explore these issues to effectively navigate the complexities introduced by these technological advancements.

## 6 Conclusion

This study developed a research model of the social networks, knowledge sharing, and job performance of PHCPs in a social media environment. It was confirmed that social networks, as indicated by degree centrality, betweenness centrality, network heterogeneity, and network tie strength, affect job performance through knowledge sharing by empirical analysis. This study not only fills in the gaps in the literature that knowledge sharing acts as an intermediary between social networks and job performance in the healthcare field but also provides many implications for promoting knowledge sharing among PHCPs and for improving their work performance.

## Data Availability

The original contributions presented in the study are included in the article/Supplementary material, further inquiries can be directed to XW, wxbtj@hust.edu.cn.
