# Supplementary material for: The association of social networks with the job performance of primary health care professionals: the mediating effect of knowledge sharing
Source: Front Med (Lausanne). 2024 Oct 3;11:1324939. doi: 10.3389/fmed.2024.1324939 (PMC11484415; doi:10.3389/fmed.2024.1324939)
Supplement: Supplementary file 1 [file Data_Sheet_1.pdf]

Appendix 1 Measurement instrument

Constructs	Items
Degree centrality	1. I reach out to many colleagues through social media to accomplish various tasks<br>2. I take the initiative to contact many colleagues through social media to consult and discuss any difficulties or problems encountered in my work.<br>3. Many colleagues often reach out to me through social media to complete various work assignments<br>4. Many colleagues reach out to me through social media to communicate and discuss any difficulties or problems they encounter at work
Betweenness centrality	5. I know a lot about my colleagues<br>6. Lots of information is conveyed to others through me<br>7. I have a strong influence within my social network
Network heterogeneity	8. My colleagues who use social media to communicate differ greatly in age<br>9. The job titles vary widely among my colleagues who use social media to communicate<br>10. The educational background varies greatly among my colleagues who use social media for communication<br>11. My colleagues who use social media to communicate have a wide range of years of experience in their organizations<br>12. My colleagues who use social media to communicate differ more in their mindset, skills, and experience
Network tie strength	13. I use social media to communicate with co-workers frequently<br>14. I communicate adequately with my colleagues in my unit when working through social media<br>15. I have a close relationship with my colleagues among my social media friends
Explicit knowledge sharing	16. I share my work reports and official documents with other co-workers through social media<br>17. I provide my manuals, methodologies, and models to other co-workers via social media<br>18. I share my learning materials with other co-workers via social media
Tacit knowledge sharing	19. I share my work experience or know-how from work with other co-workers via social media<br>20. I provide my know-where or know-whom at the request of other co-workers<br>21. I share my expertise from my education or education with co-workers via social media
Task performance	22. I always complete tasks within the time limit<br>23. I fulfill all the requirements of the job performance appraisal<br>24. I am highly productive
Interpersonal facilitation	25. I get along well with my coworkers<br>26. I support and encourage other coworkers<br>27. I cooperate effectively with my co-workers
Job dedication	28. I always support the decisions of my supervisors<br>29. I take the initiative to solve a work problem<br>30. I volunteered for extra work assignments enthusiastically
